# Eye gaze patterns reveal how reasoning skills improve with experience

**DOI:** 10.1038/s41539-018-0035-8

**Published:** 2018-10-18

**Authors:** Belén C. Guerra-Carrillo, Silvia A. Bunge

**Affiliations:** 1 Department of Psychology, University of California at Berkeley, Berkeley, CA USA; 2 Helen Wills Neuroscience Institute, University of California at Berkeley, Berkeley, CA USA

## Abstract

Reasoning, our ability to solve novel problems, has been shown to improve as a result of learning experiences. However, the underlying mechanisms of change in this high-level cognitive ability are unclear. We hypothesized that possible mechanisms include improvements in the encoding, maintenance, and/or integration of relations among mental representations – i.e., relational thinking. Here, we developed several eye gaze metrics to pinpoint learning mechanisms that underpin improved reasoning performance. We collected behavioral and eyetracking data from young adults who participated in a Law School Admission Test preparation course involving word-based reasoning problems or reading comprehension. The Reasoning group improved more than the Comprehension group on a composite measure of four visuospatial reasoning assessments. Both groups improved similarly on an eyetracking paradigm involving transitive inference problems, exhibiting faster response times while maintaining high accuracy levels; nevertheless, the Reasoning group exhibited a larger change than the Comprehension group on an ocular metric of relational thinking. Across the full sample, individual differences in response time reductions were associated with increased efficiency of relational thinking. Accounting for changes in visual search and a more specific measure of relational integration improved the prediction accuracy of the model, but changes in these two processes alone did not adequately explain behavioral improvements. These findings provide evidence of transfer of learning across different kinds of reasoning problems after completing a brief but intensive course. More broadly, the high temporal precision and rich derivable parameters of eyetracking make it a powerful approach for probing learning mechanisms.

## Psychology: Eye gaze reveals improvement in reasoning

Preparing for the Law School Admissions Test (LSAT) hones reasoning skills. A team led by Silvia Bunge at the University of California at Berkeley in the USA used behavioral and eyetracking methods to test whether, and how, studying for the LSAT would lead to improved reasoning on unrelated tasks. Young adults studying the Logic Games section of the LSAT, as compared with the Reading Comprehension section, showed improved performance on a battery of tests of reasoning. Further, eyetracking revealed a key mechanism underlying learning: increased efficiency in the ability to examine key pieces of information in a problem to arrive at a logical conclusion. Measuring eye gaze patterns during task performance can be used to track learning over time and to gain insights regarding the underlying processes of change.

## Introduction

Reasoning, the ability to solve novel problems, relies on multiple cognitive processes including relational thinking^1–3^ as well as working memory and cognitive control (e.g., refs. ^4–7^). Indeed, relational thinking is an essential component, as it allows us to form relational representations from mere percepts.^8^ Solving reasoning problems, such as those involving transitive inference, relies heavily on processes supported by relational thinking, including the ability to encode, maintain, and integrate mental relations.^1,3^ Together, these processes allow us to identify patterns and solve novel problems and are fundamental for human learning (e.g., refs. ^9,10^).

Prior research has demonstrated that reasoning can improve with targeted practice and increased task-specific expertise across the lifespan;^8,11–13^ more broadly, it has been argued that schooling hones reasoning skills.^14,15^ However, it is still unknown which aspects of reasoning contribute to improved behavioral performance. Do people become more efficient at relational thinking with experience? To address this question, we leveraged the high temporal precision and rich derivable parameters of eyetracking to index cognitive processes that may support improvements in reasoning over time.

**Fig. 1:**
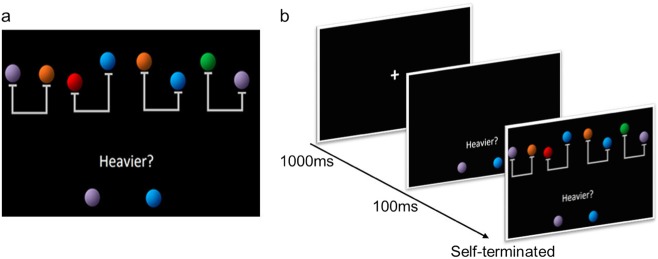
Transitive inference task. **a** Sample stimulus array, with four relations at the top and the question at the bottom. In this sample problem, participants had to encode that the blue ball was heavier than the orange one, and that the orange and purple balls were equally heavy, to determine that the blue ball was heavier than the purple one. **b** Eyetracking adaptation from refs. ^18,19^: each trial began with a fixation cross in the center of the screen (1000 ms) that cued participants to fixate on it, followed by the presentation of the question and target balls. After 100 ms, four scales would appear, only two of which were relevant to the problem. A trial ended immediately after the participant pressed a button to indicate which of the two target balls was heavier

In earlier work, our laboratory demonstrated that young adults who underwent 100 h of preparation for an exam that taxes relational reasoning (the Law School Admission Test (LSAT)) showed improvements in reasoning performance and changes in the frontoparietal network of the brain.^16–18^ Compared to a passive control group, the LSAT group improved more in accuracy and response times (RTs) on a test of transitive inference (Fig. 1a) that required the integration of novel visuospatial relations.^18,19^ Moreover, they showed a greater concomitant decrease in activation of dorsolateral prefrontal cortex,^18^ a region broadly implicated in high-level cognition (e.g., ref. ^20^). The LSAT group also showed changes in structural and resting-state functional connectivity of the frontoparietal network,^16,17^ particularly between regions implicated in relational thinking.^21,22^ Together, these findings provide evidence of experience-dependent brain plasticity as a result of practice with reasoning. However, these brain imaging results alone are insufficient to conclude which cognitive mechanisms were altered by the intervention.^23,24^

Broadly speaking, candidate mechanisms that could underlie improvements in reasoning include the ability to identify relevant pieces of information in a display (visual search)^3^ and relational thinking processes such as the encoding, maintenance, and integration of relations.^8,25^ Manipulating any of these elements have been shown to influence reasoning performance. For example, performance on a reasoning task tends to drop with an increase in the number of relations that must be integrated.^1,26^ Additionally, people tend to make incorrect deductions when individual premises contain convoluted wording, as this hinders relational encoding.^3^ Finally, drawing attention to relevant relations or segmenting a complex task to facilitate focus on single relations can improve performance, even after controlling for other task demands.^27^ These examples illustrate the point that reasoning ability relies on multiple cognitive processes.

Here we sought to determine which processes, if any, are honed with experience—both as a result of reasoning instruction/practice and more generally from repeated experience with a test (i.e., a test–retest effect). To this end, we probed changes in patterns of eye movements on a reasoning task. In the ~7 s that it takes to solve one of the problems on this task, participants make ~23 eye fixations. As such, we posited that analyzing patterns of eye movements should yield complementary insights relative to prior behavioral and brain imaging research on reasoning interventions.^12,18,28^

Participants in this study performed a transitive inference task (Fig. 1a; adapted from refs. ^18,19^) while we collected eyetracking data, both before and after they completed one of the two online LSAT preparation courses developed by Kaplan, Inc. The Logic Games course focused on reasoning about novel problems, and the Reading Comprehension course on answering questions about passages of text (see Methods for sample problems for both sections of the LSAT). Our eyetracking task requires participants to jointly consider a subset of relevant visuospatial relations depicted by balance scales (see Fig. 1). On the surface, this task bears no resemblance to the text-based problems in the LSAT curriculum. However, at a deeper level, both tax relational thinking.

We developed three gaze metrics to assess, respectively, (1) visual search; (2) a broad measure of relational thinking encompassing encoding, maintenance, and integration of relations; and (3) a more specific measure of relational integration (see Table 1 and Methods for details). Although there have been no prior eyetracking studies involving this paradigm (see refs. ^29^ for another transitive inference paradigm), our metrics were informed by eyetracking studies of analogical reasoning^22^ and matrix reasoning,^23^ as well as visual search^30^ and memory encoding.^31^ Based on these studies (see also refs. ^32,33^), we operationalized efficiency of visual search as the number of fixations needed to identify the relevant relations in a stimulus array,^30,34^ relational thinking as the total duration spent fixating the relevant relations,^35^ and relational integration, more specifically, as the number of transitions between these relations, based on the premise that increased efficiency of integrating the relations depicted in two stimuli should be manifested in looking back and forth between them fewer times.^36,37^

**Table 1:** Gaze metrics indexing processes that may support improvements in reasoning

Cognitive process	Gaze metric	Evidence supporting *H*_1_ BF_10_ ≈ *P*(*H*_1_ | data)/*P*(*H*_0_ | data)						
		*H*_1_ = POST < PRE						*H*_0_ = Group + Time
		Reasoning			Comprehension			*H*_1_ = Group × Time BF_10_
		PRE Mdn [95% CI]	POST Mdn [95% CI]	BF_10_ (% error)	PRE Mdn [95% CI]	POST Mdn [95% CI]	BF_10_ (% error)	BF_10_ (% error)
Visual search	Decrease in the number of fixations on any scale before homing in on the relevant scales	5.75 [4, 7]	5 [4, 5]	6.51* (±<0.00)	5 [4, 5.75]	4 [3, 5]	0.67 (±<0.00)	0.60 (±2.79)
Relational thinking	Decrease in the total duration of fixations on relevant relations after homing in on the relevant scales	2526.5 [1010.50, 3259.00]	1122.5 [555.99, 1854.50]	240.03*** (±<0.00)	1635.75 [1069, 2256.25]	1725 [1387.5, 1990.18]	0.30˙ (±0.02)	3.66* (±1.98)
Relational integration	Fewer saccades between the two relevant scales after homing in on the relevant scales	3 [1.75, 3.75]	2 [1, 3]	3.15* (±<0.00)	2 [2, 3]	2.5 [2, 3]	0.14˙ (±<0.00)	1.58 (±2.62)

Mdn [95% CI]: Median with 95% confidence intervals, calculated with 1000 bootstrap iterations*. H*_1_ = POST < PRE assessed with Bayesian paired single-sided *t* test. Interaction models tested with Bayesian mixed regressions. Estimations made using BayesFactor’s^53^ default Cauchy prior scale $r = \frac{1}{2}\sqrt 2$ and prior uniform probability to the models. Refer to Table S1 for specification of the models and posterior odd estimates. Approximate classification scheme for the interpretation of Bayes factors from ref. ^40^: ***Extreme evidence *H*_1_, *Moderate evidence *H*_1_ and ˙Moderate evidence for *H*_0_

**Fig. 2:**
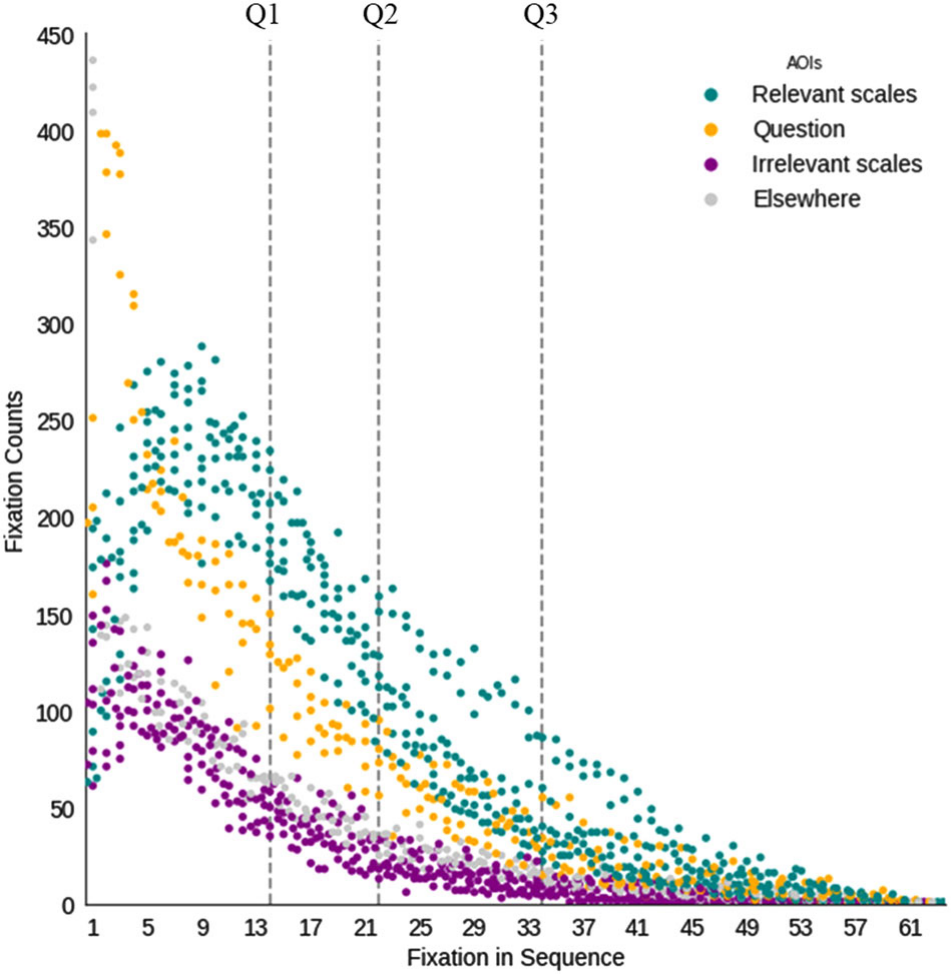
Fixations patterns on the transitive inference task. Plotting fixations during problem solving across groups and time points. Participants made a median of 22 fixations on accurately solved problems. Trials up to 64 fixations were included in the analyses (i.e., range in *x* axis). Vertical dotted lines denote quartiles of total fixations (e.g., the vertical line denoting Q3, indicates that 75% of the trials had up to 34 fixations). The colored dots represent the total number of fixations (*y* axis) from participants across both groups and time points. The colors indicate the areas of interest (AOIs) where those fixations occurred: two relevant scales (teal), two irrelevant scales (purple), and the question area (yellow). Gray points indicate fixations to points on the screen outside the AOIs

We had initially sought to use eye gaze metrics to isolate three distinct stages of task performance: visual search, relational encoding, and relational integration. However, examination of the eye gaze data (collapsed across groups and time points, Fig. 2) did not support such clear-cut stages of processing (see also ref. ^3^). First, fixations on irrelevant scales did not cease abruptly after an initial search of the array; rather, they tapered off slowly over the course of the trial (Fig. 2). As such, rather than measuring visual search as the number of fixations a participant made before looking at the irrelevant scales ever again, we identified the point in the trial at which the probability of looking at an irrelevant scale dipped below chance and the probability of looking at a relevant scale rose above chance. Second, the duration of fixations on relevant scales did not decrease over the course of a trial, as it should if this were a pure metric of relational encoding; rather, it increased (Fig. S2). This finding intimates that long fixations on a relevant scale toward the end of the trial reflect simultaneous consideration of that scale and the other relevant scale—i.e., relational integration. As such, we renamed the metric that we had previously labeled “relational encoding” to “relational thinking,” to denote the fact that it likely reflects relational encoding and maintenance toward the beginning of the trial (after preferentially looking at the relevant scales), and relational integration toward the end of the trial.

Considering the data indicated a more gradual transition from visual search to relational integration, we refined the planned gaze metrics to account for the reality of how participants solved the problems (see Methods) and then tested for effects of group and time point. Importantly, the revised metrics were unbiased with respect to effects of LSAT instruction: they were defined on the basis of data at the first time point, collapsed across groups.

Our key predictions were that reasoning instruction/practice would be associated with improved reasoning performance and efficiency in relational thinking, including the more specific measure of integration. We tested these hypothesis with behavioral and gaze data from the transitive inference task. We also assessed behavioral improvements with a composite measure of four reasoning tests, to better characterize the generalizability of the intervention.^38–40^ We considered these non-verbal measures of reasoning tests of moderate transfer, as they are very different from the word-based problems in Logic Games but have shared demands on relational thinking. The behavioral test battery additionally included assessments of working memory, planning, and selective attention, which we used to characterize the extent of transfer to untrained tasks. However, we did not anticipate improvements on these measures, given limited evidence to date of far transfer of learning in adults.^41^

Finally, we undertook an exploratory analysis to understand the cognitive mechanisms that support test–retest improvements on the transitive inference task and underlie individual differences in pre-test performance. Specifically, we examined the relationship between relational thinking, integration, visual search, and behavior.

## Results

### Improvements related to targeted reasoning instruction/practice

We quantified evidence in support of our hypotheses with Bayesian tests, permitting us to assess how likely our data are to support one model versus another using the Bayes Factor (BF_10_) and thus also quantify the strength for the null hypothesis.^42^ We used Bayesian single-sided *t* tests to gauge support for the prediction that the Reasoning group would improve in the behavioral and gaze metrics. We followed these tests with Bayesian mixed regressions to assess the probability that these changes could be attributed to reasoning practice beyond test–retest alone or subject variance. As such, we quantified the strength of evidence in favor of including the Group×Time term relative to a model containing both main effects. We modeled subject variance as a random nuisance factor, but the model design is otherwise equivalent to a 2 × 2 repeated-measures analysis of variance. We report BF_10_ (see Table S3 for detailed output) and interpret this metric in accordance with prior work:^42^ BF_10_ > 1: data provide positive evidence for the hypothesis, BF_10_ > 3: moderate evidence, BF_10_ > 10: strong evidence. The inverse applies for the null hypothesis (1/BF_10_).

#### Transfer to the composite reasoning metric

**Fig. 3:**
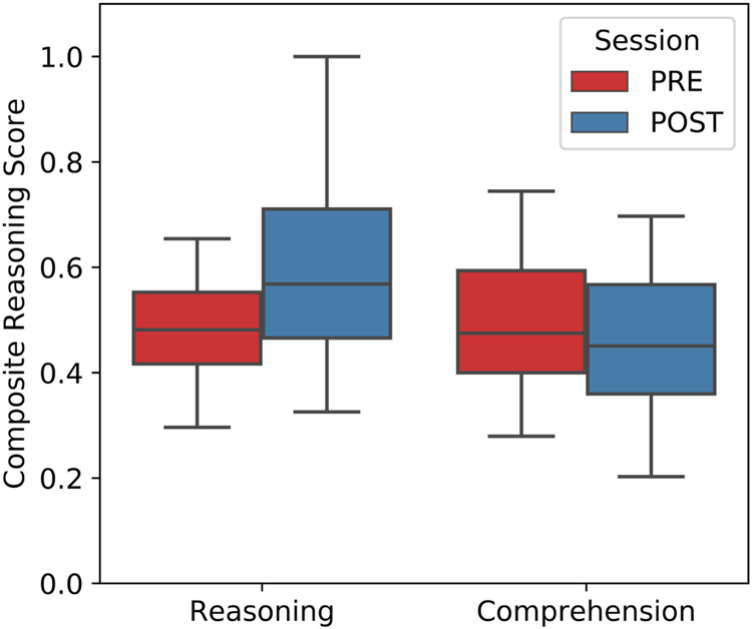
Performance on reasoning assessments. Scaled score on the composite measure of reasoning (*y* axis), before and after (*x* axis) each group completed their LSAT course. Error bars are 95% CI estimated with 5000 bootstrap iterations. **Strong evidence that the Reasoning group showed greater improvements across time points. See Table 2 for detailed statistics

The Reasoning group showed approximately a 22% improvement on this metric; by contrast, there was no evidence that performance of the Comprehension group changed between time points (Fig. 3; Table 2). The Group×Time interaction model also received strong support. Thus, according to the Bayesian analysis, there is strong evidence that the Reasoning group improved on the composite of four measures of reasoning; this was not the case for the Comprehension group.

**Table 2:** Transfer to composite reasoning measure

Subtest included	Description of subtest	Evidence supporting *H*_1_ BF_10_ ≈ *P*(*H*_1_ | data)/*P*(*H*_0_ | data)		
		*H*_1_ = POST < PRE BF_10_ (~ % error)		*H*_0_ = Group + Time
		Reasoning	Comprehension	*H*_1_ = Group × Time
				*BF*_10_ (% error)
Odd One Out	Infer rules that relate object features to identify a deviant object among 9 choices	20.78** (±<0.00)	0.63 (±<0.00)	15.06** (±3.04)
Object Reasoning	Decide whether four 2 × 2 matrices containing geometrical patterns form a sequence			
Analogical Reasoning	Apply the rule governing the relationship between three objects to a new set of objects			
Analysis Synthesis	Solve logical puzzles involving color codes representing symbolic rules			

*H*_1_ = POST < PRE assessed with Bayesian single-sided paired *t* test. Interaction models tested with Bayesian mixed regressions. Estimations made using BayesFactor’s^53^ default Cauchy prior scale $r = \frac{1}{2}\sqrt 2$ and prior uniform probabilities. See Table S1 for model specification and posterior odd estimates. Approximate classification scheme for the interpretation of Bayes factors from ref. ^40^: **Strong evidence for *H*

We also tested for transfer to the other cognitive measures in our test battery. There was no evidence that performance on these measures changed between time points in either group (Table S4). Based on these results, there was evidence of transfer from the LSAT Logic Games course to a composite score of four measures of reasoning but little evidence of far transfer from the study materials to other cognitive domains.

#### Transfer to the transitive inference task

**Fig. 4:**
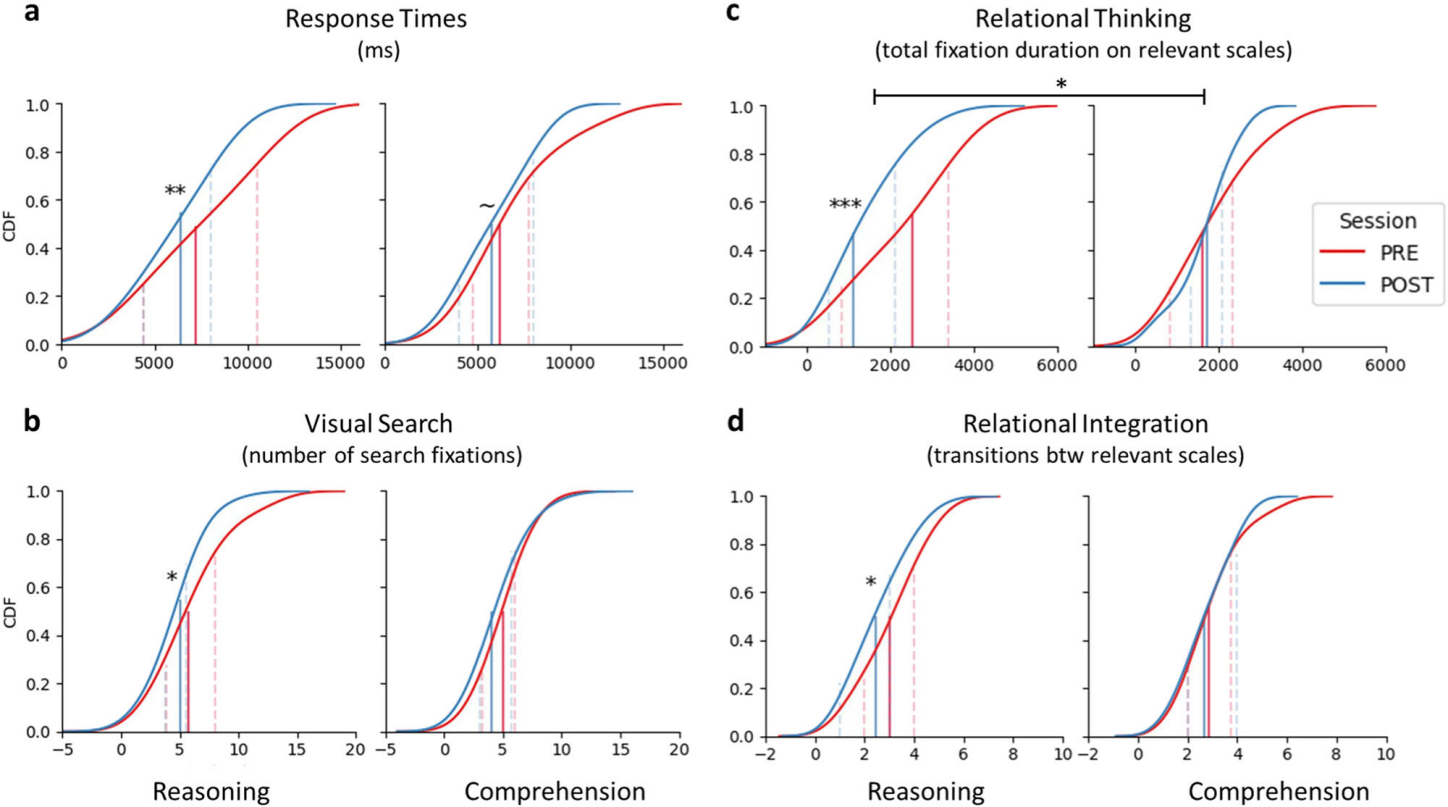
Performance and gaze metrics from the transitive inference task. Cumulative distribution functions (CDF) on each measure, for each group (left panels = Reasoning group) and time point (blue functions = post-test scores). Vertical lines denote: medians (solid), and 25th or 75th percentiles (dotted lines left or right to the median, respectively). Classification scheme to interpret Bayes factors (BF_10_) used to quantify the strength of evidence in support of the models testing: improvements across time points (i.e., POST < PRE), and differential improvements between the groups (i.e., Group×Time): ***Extreme, **Strong, *Moderate, ~Anecdotal. Refer to Table 1 for detailed statistics

Given that accuracy was at ceiling already at pre-test (Fig. S1) for this sample, unlike the sample in our prior study,^18^ we focused exclusively on RTs on correct problems as a measure of performance. The data provided strong evidence in support of the hypothesis that the Reasoning group would become faster at accurately solving the problems between time points (BF_10_ ≈ 27.41 ± < 0.00, Δ ≈ 18%). There was positive, albeit weaker, evidence that the Comprehension group also improved (BF_10_ ≈ 2.18 ± < 0.00). Although the data provided support for the model containing the Group×Time term (BF_10_ ≈ 8.30 ± 4.48%), the strongest model included only the effect of Time (BF_10_ ≈ 39.24 ± 2.41%). Thus, although the evidence of improvement was stronger for the Reasoning group, both groups got faster at solving the task between time points (Fig. 4a).

#### Gaze metrics

We predicted that reasoning instruction would lead to improved efficiency of relational thinking, including changes in relational integration. For the Reasoning group, the gaze data provided moderate support for the model stipulating changes in the selective measure of relational integration, and extreme evidence in favor of the model testing improvements in the broader measure of relational thinking (for these and subsequent results, see Table 1; Fig. 4b–d). By contrast, for the Comprehension group, there was moderate evidence in favor of the null model for both metrics, suggesting that their pre- and post-test scores are comparable. When considering whether the changes in relational thinking in the Reasoning group were greater than in the Comprehension group, we find that the data are 3.66 times more likely under the model including the Group×Time term compared to the model testing only the main effects. Indeed, the interaction model was best supported by the relational thinking data (BF_10_ ≈ 7.27 ± 1.51%). Conversely, there was moderate evidence against including the interaction term to model the relational integration data (BF_10_ ≈ 0.17 ± 2.32%). Thus there is moderate evidence that reasoning instruction/practice led to improved efficiency of relational thinking.

We also tested for improved efficiency of visual search. For the Reasoning group, the data provided moderate support for the model stipulating increased search efficiency; this was not the case for the Comprehension group. However, the data did not support the Group×Time model: rather, there was moderate support for the main effect of Time (BF_10_ ≈ 4.01 ± 1.14%). These results suggest that changes in visual search were likely due to individual differences and test–retest effects present across both groups, rather than to an effect of reasoning instruction/practice.

### Individual differences in performance and test–retest improvements on the transitive inference task across the full sample

We first assessed the relationships among the gaze metrics at pre-test with Kendall’s tau Bayesian correlations. At pre-test, relational thinking was strongly correlated with both visual search (*τ* = 0.40, BF_10_ ≈ 161.67) and relational integration (*τ* = 0.64, BF_10_ ≈ 4.742e + 6), but the latter two metrics were only moderately correlated with each other (*τ* = 0.29, BF_10_ ≈ 6.41). Similarly, change in relational thinking correlated strongly with changes in relational integration (*τ* = 0.53, BF_10_ ≈ 24534.15) and moderately with changes in visual search (*τ* = 0.29, BF_10_ ≈ 6.61); by contrast, there was no evidence that changes in visual search and relational integration were correlated with one another (*τ* = 0.19, BF_10_ ≈ 0.91). These results provide evidence that greater efficiency of relational thinking was related to both visual search and relational integration, which in turn were separable components of relational reasoning.

Next, we used Bayesian regression models to determine which gaze metric(s) could best explain changes in RTs between time points across the full sample. The models included LSAT group as a nuisance variable and RT changes as a dependent variable. The predictors included one or more of the gaze metrics, scaled to the same units (deviations from the grand mean). We found the strongest evidence in favor of a model that included changes in relational integration, relational thinking, and visual search as predictors of change in RTs (BF_10_ ≈ 27.75 ± 0.85%). Together, these metrics accounted for ~35% of the variance in RT reduction. In simpler models testing individual gaze predictors of changes in RTs, there was strong evidence in favor of relational thinking (BF_10_ ≈ 20.11 ± 1.70%), which accounted for ~23% of the variance in RT reductions (*β* = 0.72). By contrast, there was mild evidence for visual search (BF_10_ ≈ 2.83 ± 0.79%, *R*^2^ = 0.14, *β* = 0.25) and no evidence in favor of the more specific measure of relational integration (BF_10_ ≈ 0.53 ± 1.52%). The fact that the relational thinking metric was a continuous, time-based measure rather than a frequency count (unlike visual search and relational integration) cannot explain why it was more predictive of change in RTs than visual search: in a follow-up analysis, we found that a continuous measure of visual search, total duration, explained even less of the variance than the number of search fixations (BF_10_ ≈ 0.20 ± 1.42%, *R*^2^ = 0.05, *β* = 0.19). Overall, these results point to relational thinking as a key facet of practice-related improvements in transitive inference task performance.

## Discussion

Which cognitive mechanisms underlie improvements in relational reasoning? We sought to address this question by examining improvements related to targeted reasoning instruction and practice with the Logic Games section of the LSAT and general test–retest improvements across two time points by also considering the changes in the group who prepared for the Reading Comprehension section of the LSAT. Our key prediction was that practicing solving word-based logic problems would lead to improved performance on visuospatial tests of reasoning, as well as improved efficiency of relational thinking on a visuospatial transitive inference task, as measured via gaze metrics. We additionally tested whether either group exhibited increased efficiency of visual search.

We found evidence that reasoning instruction/practice led to improved performance on a composite of four measures of reasoning. On the surface, these measures of reasoning did not resemble the LSAT problems but rather shared a deeper commonality of demands on relational thinking. Thus these results provide evidence of moderate transfer from one type of reasoning practice to other reasoning tests.

Additionally, we found evidence that reasoning instruction/practice led to increased efficiency on our ocular measure of relational thinking. By contrast, there was no compelling evidence that the changes in visual search or the specific metric of relational integration could be attributed to the intervention. Thus we conclude that reasoning instruction/practice predominantly honed the ability to encode and maintain several mental relations in mind.

Although reasoning/instruction affected relational thinking on the transitive inference task, it did not yield a benefit in terms of behavioral changes on this task over and above a test–retest effect. This discrepancy illustrates the idea that gaze metrics can pinpoint changes in specific cognitive processes even if the behavioral measures administered in the study are insufficiently sensitive. Similar arguments have been made with regard to brain imaging studies examining the effects of an intervention^43^ or predicting future behavior.^44^

Having found evidence that the Reasoning group improved on the composite reasoning measure, we sought to characterize the extent of transfer to other cognitive domains. In our prior study,^18^ we did not observe transfer of reasoning instruction/practice to individual measures of matrix reasoning, rule induction, working memory, or processing speed. Here we examined transfer to composite measures of various cognitive abilities, rather than individual tests, as a more robust test of transfer.^40^ We found moderate evidence in favor of the null hypothesis—i.e., no change—for measures of planning, working memory, selective attention, and verbal comprehension. Taking together the results from both studies, there is—as predicted based on prior intervention studies^41,45^—no evidence of far transfer from LSAT practice.

Finally, we adopted an individual differences approach to understand the processes that support test–retest improvements and performance on the transitive inference task. At pre-test, relational thinking was strongly correlated with both visual search and relational integration, but these two metrics were only weakly correlated with each other. This pattern of results was also obtained for correlations among the change scores for these metrics. These findings suggest that there could be temporal overlap between visual search and the early stages of relational thinking (initial encoding of scales), whereas relational integration may overlap with the later stages (maintenance of the relevant relations). It is also plausible that our metric of relational integration includes a verification or checking process that occurs after relational integration but prior to responding. Similar ocular behaviors related to confirming an answer have been observed in cognitive assessments of planning.^46^

Across the entire sample, we found evidence that RT reductions were associated with improved efficiency of relational thinking and visual search but that relational thinking was likely the strongest driver of change. This finding, along with the pattern of correlations among gaze metrics, suggests that these gaze metrics capture at least partially separable cognitive processes—and that each contributes differentially to improved performance. Indeed, even when accounting for improved attentional control underlying visual search, changes in relational thinking are still a critical predictor of improved reasoning performance.

Although this study leverages eyetracking measures in a novel way to provide insights regarding learning mechanisms, there are several limitations to consider. First, while we have evidence that practicing Logic Games is associated with gains in other measures of reasoning, we lack strong evidence that Logic Games performance itself improved after the 6-week online course, as measured via one brief (8-min) problem set administered at pre-test and two at post-test. This outcome contrasts with the improvements we found in our laboratory’s prior study,^18^ which differed from the current study in multiple ways. First, changes in LSAT performance in the previous study were assessed with full-length practice exams, which included 4 problem sets (35 min each) for Logic Games as well as for Reading Comprehension. Although the problems we had selected are considered of medium difficulty and test common question types, the strategies taught in the LSAT course may not have been particularly useful for solving the specific problems we selected.

Second, although participants rated both courses as effective, the online course format—while ideal from an experimental standpoint, as it enabled us to compare two separate but similarly structured courses—may not have been an ideal learning platform. Additionally, those participants who did not plan to take the LSAT in the near future are likely to have devoted less time to their study than they would have otherwise; with our self-report measure, we do not know for certain how many hours they spent on the paper-and-pencil practice problems.

Finally, there may have been a synergistic effect in the previous study of studying for all sections of the LSAT together (Logic Games, Analytical Reasoning, and Reading Comprehension) and spreading the course over 3 months as opposed to 6 weeks. However, despite the lack of improvement on our brief Logic Games assessment, we contend that we can meaningfully assess effects of this experience on other assessments that tap overlapping skills.

Another limitation of the study is that pre-test accuracy on the transitive inference task was at ceiling, in contrast with our prior study.^18^ This difference likely reflects differing sample characteristics. The task was sensitive to RTs in this study, but the two groups sped up to a similar degree. We can only speculate that, if the task had been more difficult for these participants, we would have had an opportunity to observe a differential effect of Reasoning and Comprehension courses on accuracy.

A final limitation is that recruitment and retention were challenging. The study required students who were inexperienced with the LSAT and were willing to commit to studying for only one section—a requirement that likely dissuaded students who sought to take the LSAT immediately. Additionally, the study required a serious time commitment for undergraduates who already had a full course load. However, considering that there were similar levels of attrition in the Reasoning and the Comprehension groups, we are still able to draw meaningful conclusions about the effects of reasoning practice.

To conclude, our study highlights the utility of eyetracking for probing the mechanisms underlying real-world learning. The gaze metrics revealed that changes in relational thinking contributed to improved reasoning performance, beyond changes in supporting attentional processes. The high temporal resolution of the eyetracker provides a more detailed window into the series of rapid computations and highly interactive processes that underlie reasoning^27^ than is possible with neuroimaging or behavioral methods alone. Beyond elucidating mechanisms of plasticity, then, the metrics and observations reported here could inform future research on the thought processes that unfold during reasoning. Finally, the combined use of eyetracking with neuroimaging methods could provide unique insights into the brain mechanisms that support cognitive functioning and learning and sources of individual differences therein.

## Methods

### Participants and eligibility

We recruited college students planning to take the LSAT within 1 year. Inclusion criteria included being native English speakers; at least 18 years; normal/corrected vision; and no history of psychiatric disorders, learning disabilities, or prior LSAT experience. Participants were assigned pseudo-randomly to study for one of these two sections of the LSAT, the Logic Games or the Reading Comprehension section. The first quarter of participants were assigned to a group at random, whereas we distributed the rest to match the groups on age, gender, reasoning, working memory, and LSAT performance (Table S1). We collected data from 2015 to 2017, following the semester structure of UC Berkeley: Spring (January–May) 2015, Summer (June–August) 2015, Fall (August–December) 2015, Spring 2016, Summer 2016, Fall 2016, and Spring 2017.

Ninety-five participants completed the pre-tests, and 49 completed the LSAT course and post-tests. We excluded two of these participants because they failed to study for their assigned course. Participants in our final sample did not differ from those who only completed one time point on either cognitive performance or demographic variables. The final sample who prepared for the Logic Games section of the LSAT included 23 students (14 females, mean age 21.55 years). The final sample who prepared for the Reading Comprehension section of the LSAT included 24 students (13 females, mean age 21.88 years). Levels of attrition did not differ significantly between the groups (*χ*² = 0.01, *p* = 0.93). For analyses involving the transitive inference task, we excluded two subjects from each group for having >60% of trials missing valid fixation data, and one subject from the Reasoning group for having performance below chance levels (20% accuracy, chance was 50%). The research was approved by the Committee for the Protection of Human Subjects at the University of California, Berkeley. Written informed consent was obtained from all participants.

### Summary of procedures

Before and after studying for the LSAT courses, participants completed a battery of nine online cognitive assessments,^47^ followed by an in-person testing session. Participants were blind to their LSAT group at pre-test, and the experimenters carrying out the testing sessions were blind to the group assignment at both time points.

During the laboratory sessions, we recorded gaze data from participants while they completed a transitive inference task, followed by two tests of inductive reasoning. Data from the transitive inference task is the subject of the current investigation. After finishing the eyetracking tasks, participants completed a standardized test of reasoning termed Analysis Synthesis (Woodcock–Johnson Battery III^48^), LSAT sample problems, and a survey. The survey included demographic and ocular health questions, questions regarding prior experience with the LSAT, and at post-test, questions about the participant’s experience with their LSAT course. The order of tests was the same at both time points.

### LSAT courses

Participants studied for either the Logic Games or Reading Comprehension section of the LSAT with a commercially available online course (Kaplan, Inc.) for 6 weeks. The courses were similar in critical ways. Both courses included six lessons, each consisting of online videos and homework practice problems designed to help improve timing and increase mastery with different question types. Both courses featured the same instructors in the online videos, who explained problem-solving strategies and had students practice those skills with real LSAT problems. Students had access to the online course materials and were given a companion workbook that included practice problems.

We requested that participants (1) study only for the LSAT section we assigned to them, (2) complete all six lessons of the course within 7 weeks (approximately one lesson/week), and (3) space their practice (i.e., study every other day, three times per week), in keeping with prior work showing that spacing practice promotes learning^49^ and transfer effects.^50^ We chose these practice intervals so that students could incorporate their LSAT courses more easily with their typical school schedules. Participants reported having complied with these instructions and that they had completed on average one lesson per week (range = 0.5–2 lessons) and studied their course for on average 24 h (first quartile: 16; third quartile: 36). Both groups reported similar studying times (median = 24 h for each group).

The Logic Games section involves solving word problems that contain many rules that must be integrated to find the correct answer (sample problems: https://www.lsac.org/jd/lsat/prep/analytical-reasoning). The preparatory course for this section instructed on strategies such as organizing relational information into sketches to minimize the amount of information one needs to remember, as well as to facilitate deductions, rule abstractions, and correct rule application.

The Reading Comprehension section involves reading long passages and answering multiple choice questions based on relevant information in the passages (sample problems: https://www.lsac.org/jd/lsat/prep/reading-comprehension). The preparatory course for this section involved learning strategic reading techniques, such as finding keywords based on the passage questions and annotating main ideas on the passages to minimize working memory demands.

Participants in the two groups found their respective courses relatively effective and enjoyable, with no differences between groups (Table S2). However, we measured the effectiveness of the LSAT courses with short Logic Games and Reading Comprehension problem sets that participants completed in the laboratory and found little evidence of the effectiveness of the mini-courses in improving performance on either section (S1).

### Eyetracking apparatus and procedures

We recorded binocular gaze data from participants completing a transitive inference task using Tobii T120 Eye Tracker (17-inch monitor, 1280 × 1024 pixel resolution). We sampled at a temporal resolution of 120 Hz, with participants sitting at 60 cm from the eyetracker camera. We took several precautions to collect high-quality ocular data following recommendations from.^51^ Furthermore, participants reported that they did not suffer from medical conditions or used medication that could affect ocular behaviors. We used Presentation® software (v. 18.0, Neurobehavioral Systems, Inc.) to present the task stimuli and the Tobii Eye Tracker Extension for Presentation v1.1^52^ to synchronize the timing of the stimulus presentation and ocular events.

### Transitive inference task

In the transitive inference task (adapted from a task we had developed previously for functional magnetic resonance imaging research;^18,19^ Fig. 1), participants saw four balance scales, each one with two color balls. Based on the relations shown by the scales, participants needed to infer the relative weights of two target balls. To solve the problems correctly, it was necessary to integrate the relationship shown by two of the four scales (i.e., the relevant scales). Participants completed 60 of these problems, divided into two blocks of 30 trials. We recalibrated the eyetracker during the short break between blocks.

We minimized potential confounds in gaze patterns by controlling for features that could impact visual saliency and subjects’ expectations as to where the relevant scales were likely to appear and which balls were likely to be relevant. We changed the position of the relevant scales across trials, and the program selected the color of the five balls at random from a set of six colors, which were all matched in luminance. Additionally, we biased the participant’s first fixation to the question area by first presenting the question alone for 100 ms and then adding the four scales (see trial sequence in Fig. 1b). We staggered the stimulus presentation in this way in an effort to encourage participants to begin the task by searching for the relevant relations and then proceed to integrating them.

### Behavioral outcome measures

We examined changes in RTs and accuracy (proportion of trials answered correctly). Performance did not vary as a function of the spatial arrangement of the scales (e.g., the position of relevant scales) or the number of scales showing inequalities (Fig S1). Thus we did not include these factors in our analyses in favor of maximizing the statistical power to assess our hypotheses.

Given that pre-test RTs were highly positively skewed (sk *=* 4.55), we trimmed outlier trials falling on the long end of the tail (i.e., Q3 + 1.5 × IQR) to minimize bias in our gaze analysis that could result from including the highly variable fixation durations that could occur on these atypically long trials. Outlier trials were identified separately by subject, time point, and block, to retain individual differences in performance. Approximately 5% of trials were trimmed owing to outlier RTs from each group per time point.

### Gaze preprocessing and outcome measures

We classified gaze data into fixations using a standard dispersion-based algorithm adapted from ref. ^53^ allowing a maximum dispersion of 35px over a 100 ms window (see details in Section S2). Participants had a median of 22 fixations on correct trials. Our analysis included only trials with at least three valid fixations, under the assumption that this is the minimum number of fixations needed to solve the problem, with a maximum of 64 fixations (i.e., Q3 + 1.5 × IQR) to minimize the bias that those outlier trials could induce.

We assigned an area of interest (AOI) label to the fixations. The AOIs included each of the four scales (two relevant and two irrelevant scales) and the area where the target balls and question appeared. We used these labeled fixations to calculate the number of gaze transitions between different AOIs. For instance, a fixation on “Relevant Scale 1” followed by a fixation on “Relevant Scale 2” was coded as one transition between the relevant scales. We refer to these events as transitions because we were primarily concerned with measuring how often fixations shifted between two different scales; we ignored, at most, one fixation that may have occurred elsewhere between those two target fixations.

We used the transitions and fixation data from each trial to derive three gaze outcome measures (Table 1), informed by an analysis of fixation sequences performed across groups and time points (Fig. 2). To compute the gaze metrics, we first marked the point at which it became more probable that a participant had homed in on the relevant scales during a trial. For each trial, and on an individual subject basis, we measured that point in the trial by calculating the empirical probability that the number of fixations on irrelevant scales was below chance (25%) and that the number of fixations on relevant scales was greater than chance. We estimated these probabilities with a sliding window that evaluated 20% of the fixations at once (min. size 4, max. size 8 fixations). This approach enabled us to capture a common pattern of fixations (Fig. 2), whereby participants began to preferentially fixate on the relevant scales after a certain point in the trial. Accordingly, the visual search metric constitutes the number of fixations the participant made on any scale prior to that point, and we indexed relational thinking as the duration of fixations on relevant scales occurring after that point. We additionally computed a more specific metric of relational integration as the number of saccades between the two relevant scales.

### Composite reasoning measure and other transfer tasks

Three subtests included in the composite reasoning measure (Table 2) were part of a larger battery of nine online assessments, which included tests of selective attention, planning, and working memory (Table S4). These tests were developed by the Cambridge Brain Sciences Laboratory (http://www.cambridgebrainsciences.com) as an online adaptation of assessments designed and validated at the Medical Research Council Cognition and Brain Sciences Unit.^47,54^

Task difficulty in all the assessments was adaptive as a function of performance. Performance metrics differed between the tasks (e.g., a maximum level achieved versus total correct responses), so we standardized the scores after removing outlier scores (i.e., scores that deviated >3 S.D. away from the grand pre-test mean). Using this normalized dataset, we created composite measures of reasoning, planning, and working memory by averaging performance across related assessments. Composite measures provide a robust test of transfer^38,40^ and help minimize the number of statistical tests necessary. We derived these composite measures with a theory-driven approach, given that factor analytic methods were not appropriate for our sample size. For the reasoning measure, we averaged the standardized scores from the Analogical Reasoning, Object Reasoning, and Odd One Out tests, as well as the Analysis Synthesis test administered in the laboratory.

### Statistical analysis

We used Bayesian models to quantify the strength of evidence supporting the model that tested a given hypothesis in question, as described in the Results section. For all analyses, we used participant’s median scores on the measure of interest and uniform distribution of prior probabilities with default Cauchy prior scales from the BayesFactor R package.^55^ The sample size was sufficient for the Bayesian analysis performed. For traditional hypothesis testing analysis, the sample size is sufficient to test for the effects of reasoning practice between time points with a power of 0.86 and an alpha criterion of 0.05, as well as a Group×Time interaction effect with a power of 0.73 and an alpha criterion of 0.05.

### Code availability

We used custom scripts written in Python (v3.6) to preprocess and calculate gaze outcome metrics and R (v3.2) to perform the Bayesian analysis. The code and instructions can be found in the Open Science Framework repository, https://osf.io/hkzgw/?view_only=8f4749510a2f44ef86fea154e9f6e9c4.

## Electronic supplementary material

Supplemental Material

## Data Availability

The dataset generated and analyzed during this study is accessible in the Open Science Framework repository, https://osf.io/hkzgw/?view_only=8f4749510a2f44ef86fea154e9f6e9c4

## Electronic supplementary material

**Supplementary information** accompanies the paper on the *npj Science of Learning* website (10.1038/s41539-018-0035-8).
