# Supplementary material for: Eye gaze patterns reveal how reasoning skills improve with experience
Source: NPJ Sci Learn. 2018 Oct 18;3:18. doi: 10.1038/s41539-018-0035-8 (PMC6220245; doi:10.1038/s41539-018-0035-8)
Supplement: Supplementary file 1 — Supplemental Material [file 41539_2018_35_MOESM1_ESM.docx]

# **Eye gaze patterns reveal how reasoning skills improve with experience: supplementary information**

**Sample**

**Table S1. Participant Information**: Demographic information and variables used to match the groups at pre-test.

**LSAT manipulation**

**Section S1. Improvements in LSAT performance**: Results of analyses examining improvements in performance on the LSAT sections based on problem sets completed in the lab.

**Table S2. LSAT self-report data and strategy use ratings:** Perceived level of improvement and changes in strategy use when solving LSAT problems administered in the lab.

**Transitive inference task: gaze and behavioral metrics**

**Fig S1. Accuracy and response times (RTs) based on problem type**: Plot showing that behavioral performance did not differ at pre-test as a function of the placement of the relevant scales in the stimulus array, or whether they involved inferences between two scales representing inequalities (e.g., the green ball is heavier than the yellow ball), or one representing an equality (e.g., the green and yellow balls are equally heavy) and the other an inequality. Figure also shows that accuracy was at ceiling at pre-test.

**Section S2**. **Gaze data preprocessing and fixation detection algorithm:** Describes algorithm used to preprocess gaze data and identify fixations. Provides support to methods section.

**Fig S2. Count and median duration of fixations on relevant scales on the transitive inference task.** Plotting fixations on relevant scales across groups and timepoints.

**Bayesian Analysis**

**Table S3. Posterior odds and model comparison:** Gaze metrics and composite reasoning score. Provides supporting information to Tables 1 and 2 in the main manuscript.

**Table S4. Performance on the behavioral assessments:** Test of transfer to other cognitive domains including working memory, planning, selective attention, and verbal fluency.

**Sample**

**Table S1.** Participant Information and variables used to match groups at pretest

|  | **Reasoning group**  Logic Games Course  *n* = 23 | **Active control**  Comprehension Course  *n* = 24 | Pairwise comparisons  *t or Χ^2^* |
| --- | --- | --- | --- |
| Females | 14 | 13 | *0.12* |
| Age | 21.55 (3.96) | 21.88 (4.93) | 0.25 |
| WJ III Analysis Synthesis | 29.39 (1.90) | 30.43 (2.61) | 1.55 |
| Digit Span | 7.00 (2.34) | 6.92 (1.18) | -0.16 |
| Spatial Span | 6.39 (1.53) | 6.38 (0.97) | -0.04 |
| Logic Games sample problems | 1.74 (1.25) | 1.58 (1.10) | -0.45 |
| Reading Comprehension sample problems | 2.61 (1.28) | 2.62 (1.12) | 0.05 |
| LSAT score (T1 practice exam) | 145 | 147 | -0.83 |

Mean and SDs. *t* or *X^2^* values reported for the variables the groups were matched on at T1 (p’s > 0.54, Analysis Synthesis p=0.14). Performance on assessments as raw scores. The maximum score on the sample LSAT Logic Games and Reading Comprehension problems are six points. LSAT scores are based on the full practice exam administered at T1 and on the standard 120-180 LSAT point scale.

**LSAT manipulation**

**S1. Improvements in LSAT performance**

We tested for changes in performance on a set of Logic Games and Reading Comprehension problems administered in the lab at each timepoint^[[1]](#footnote-1)^. Each set consisted of 6 problems. An additional set of 6 questions per section was administered at post-test^[[2]](#footnote-2)^. Participants had up to eight minutes to answer each problem set.We found no significant effect of Time, Group, or their interaction on performance on the Reading Comprehension problems (all *p* > 0.36) or the Logic Games problems (all *p* > 0.14) administered at both timepoints. On the new set of problems administered only at post-test, the Comprehension group performed better on the Reading Comprehension problems (*t*(42) = 2.07, *p* = 0.05), and incorporated the strategies taught in their course more when solving these problems (Table S2). However, the groups did not differ in performance on the Logic Games problems (*t*(42) = -0.66, *p* = 0.51). Although participants in both groups reported after the post-test assessment that they thought they had improved most on the section they had studied (Table S2), it was not the case that the Logic Games group improved more than the other group on Logic Games – at least, not on the basis of the limited number of LSAT problems administered in the laboratory.

Secondly, although participants rated both courses as effective, the online course format – while ideal from an experimental standpoint, as it enabled us to compare two separate but similarly structured courses – may not have been an ideal learning platform. Relatedly, our participants completed the online course as part of a study rather than being recruited based on their enrollment in an actual LSAT course. As such, students might have devoted less time to their study than they would have otherwise; with our self-report measure, we do not know for certain how many hours they spent.

Finally, there may have been a synergistic effect in the previous study of studying for all sections of the LSAT together (Logic Games, Analytical Reasoning, and Reading Comprehension), and spreading the course over 3 months as opposed to 6 weeks.

**Table S2.** LSAT self-report data and strategy use ratings

|  | **Reasoning group**  Logic Games Course  *n* = 23 | **Active control**  Comprehension Course  *n* = 24 |
| --- | --- | --- |
| Rating of course effectiveness | 4 (“effective”) | 4 (“effective”) |
| Rating of course enjoyment | 3 (“neutral”) | 3 (“neutral”) |
| Perceived improvements on Logic Games | 4 (“stronger performance”) | 3 (“no change”) |
| Perceived improvements on Reading Comprehension | 3 (“no change”) | 4 (“stronger performance”) |
| % change in Logic Games strategy-use | 11% | 11% |
| % change in Reading Comprehension strategy-use | 25% | 56% |

Median of self-reported enjoyment, course effectiveness, and perceived improvement on a 5-point Likert scale, showing in parenthesis the answer choice equivalent to the numerical rating. The original scale of “perceived improvements” was recoded from a range of [-2, +2] to [1, 5] on this table to be consistent with the other measures. Use of LSAT strategies could amount to 10 points each. Showing the percent change for each section, calculated using the median score at each timepoint. Scoring of strategies available upon request.

**Transitive inference task: gaze and behavioral metrics**


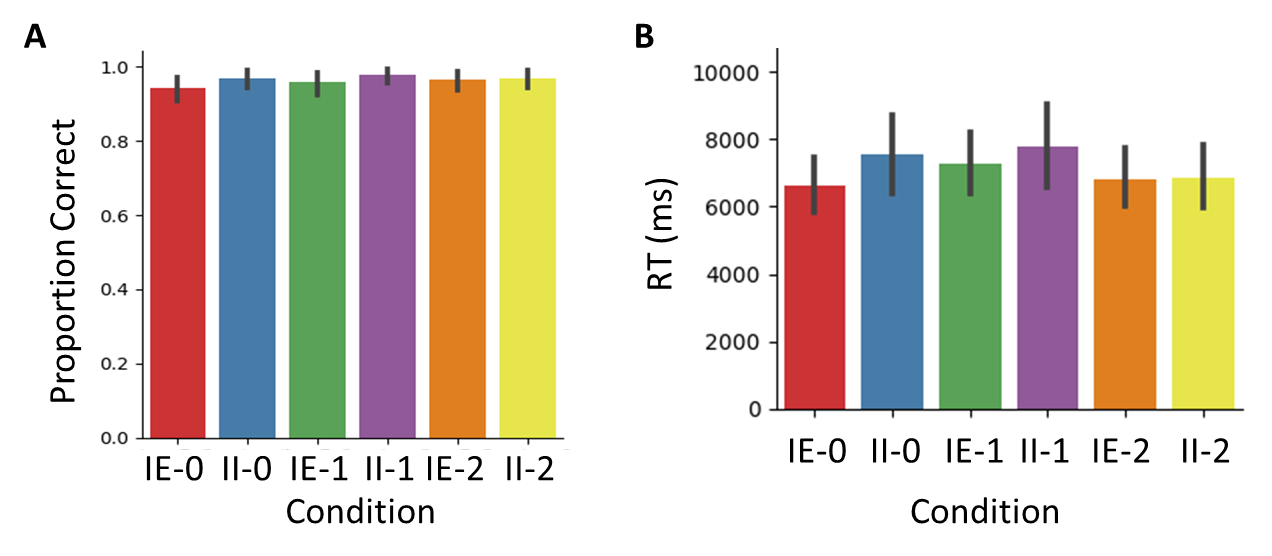


**Fig S1. Effect of problem type on behavioral performance on the transitive inference task at pretest.** The order in which relevant relations appeared (i.e., 0-2 scales apart) or whether the question involved integrating between inequalities (II) or between an inequality and an equality (IE) did not significantly impact either a) proportion of correct responses, or b) RTs on accurate trials. All error bars are 95% C.I estimated with 5000 bootstrap iterations

**S2. Gaze data preprocessing and fixation detection algorithm**

We used custom scripts written in Python v3.6 to preprocess the gaze data and identify fixations. We classified gaze data points into fixations using a dispersion-based algorithm ^1^, such that in a 100ms window the gaze position could not exceed a radius of 35 pixels (1° visual angle) to be considered part of the same fixation. That distance has been previously identified as a reliable threshold for accurate fixation classification ^2^ and is sufficient given the size of our stimuli. Single gaze points that deviated from the distance threshold were considered part of the same ongoing fixation as long as the next legitimate gaze measure returned to the accepted threshold. Gaps between gaze data points of less than 40ms were ignored, as it is physiologically unlikely that a separate fixation or blink occurred during that time; it is more likely that those gaps represent an artifact of the apparatus ^3^. Both groups showed a reduction in total fixation times at T2 (*t*(44) = -3.52, *p* < 0.01), but the groups did not significantly differ on total fixation times at either timepoint (*p* > 0.2).


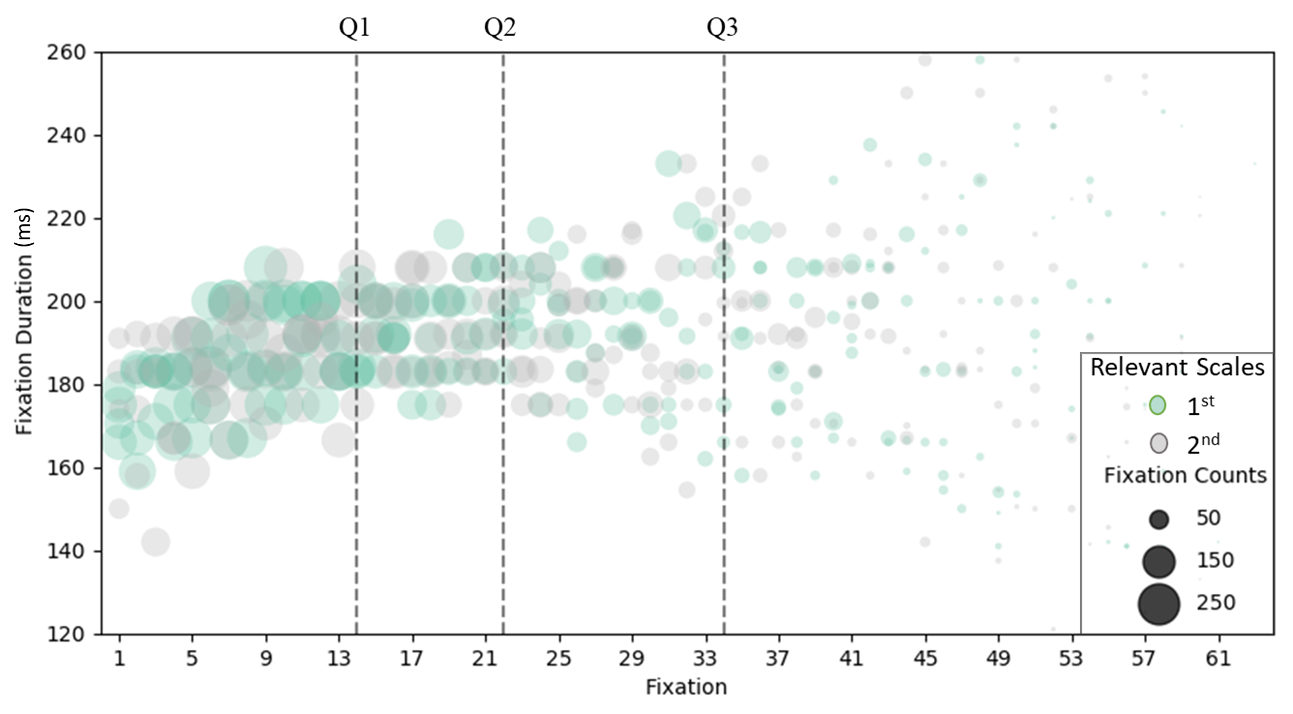


**Fig S2. Count and median duration of fixations on relevant scales on the transitive inference task.** Plotting fixations on relevant scales across groups and timepoints on accurate problems. Trials up to 64 fixations were included in the analyses (i.e., range in x-axis). Vertical dotted lines denote quartiles of total fixations (e.g., the vertical line denoting Q3, indicates that 75% of the trials had up to 34 fixations). Median fixation duration (y-axis) and total fixation count (size of circles) calculated across groups and timepoints. The colors indicate whether fixations occurred on the first (i.e., most-left during a trial; green) or second (gray) relevant scale.

**Bayesian Analysis**

**Table S3**. Posterior odds and model comparison: gaze metrics and composite reasoning score

| **Metric** | **Models** | **P(M)** | **P(M\|data)** | **BF_M_** | **BF_10_** | **error %** |
| --- | --- | --- | --- | --- | --- | --- |
| Visual search | Null model (incl. Group, Time, Subject) | 0.50 | 0.63 | 1.73 | 1.00 |  |
|  | Group x Time | 0.50 | 0.37 | 0.60 | 0.60 | 2.79 |
| Relational Thinking | Null model (incl. Group, Time, Subject) | 0.50 | 0.21 | 0.27 | 1.00 |  |
|  | Group x Time | 0.50 | 0.79 | 3.66 | 3.66 | 1.98 |
| Relational Integration | Null model (incl. Group, Time, Subject) | 0.50 | 0.40 | 0.68 | 1.00 |  |
|  | Group x Time | 0.50 | 0.60 | 1.58 | 1.58 | 2.62 |
| Composite Reasoning | Null model (incl. Group, Time, Subject) | 0.50 | 0.06 | 0.07 | 1.00 |  |
|  | Group x Time | 0.50 | 0.94 | 15.06 | 15.06 | 3.04 |

Estimations made using BayesFactor’s ^4^ default Cauchy prior scale $r=\frac{1}{2} \sqrt{2}$

**Table S4**. Performance scores on the online behavioral assessments.

| Composite Measure | Subtest | Description | Evidence supporting *H*_1_  *BF*_10_ ≈ *P(H_1_* \| data*) /* *P*(*H*_0_ \| data*)*  *H_1_* = *POST < PRE* | | | | | |
| --- | --- | --- | --- | --- | --- | --- | --- | --- |
|  |  |  | Reasoning | | | Comprehension | | |
|  |  |  | PRE  *M*  *(± S.D)* | POST  *M*  *(± S.D)* | *BF_10_*  *(%error)* | PRE  *M*  *(± S.D)* | POST *M*  *(± S.D)* | *BF_10_*  *(%error)* |
| Planning | Spatial Slider | Reconfigure numbered shapes in ascending order using as few moves as possible | 0.43 (0.19) | 0.46 (0.20) | 0.25**˙** (±<0.00) | 0.49 (0.20) | 0.55 (0.23) | 0.50 (±<0.00) |
|  | Hampshire Tree Task |  |  |  |  |  |  |  |
| Working Memory | Digit Span Forward | Hold in mind strings of digits or spatial locations and recall them in the order in which they had appeared | 0.53 (0.16) | 0.55 (0.17) | 0.20**˙** (±<0.00) | 0.59 (0.21) | 0.56 (0.20) | 0.34**˙** (±0.01) |
|  | Spatial Span Backward |  |  |  |  |  |  |  |
| Selective Attention | Feature Match | Decide whether all the geometrical elements in a visual array were identical | 0.52 (0.24) | 0.57 (0.24) | 0.6 (±0.01) | 0.56 (0.26) | 0.64 (0.24) | 0.99  (±<0.00) |
| Verbal Comprehension | Grammatical Reasoning | Make speeded judgments as to whether a statement correctly describes a pair of objects | 0.38 (0.20) | 0.33 (0.25) | 0.19**˙** (±<0.00) | 0.38 (0.25) | 0.41 (0.30) | 0.37**˙** (±<0.00) |

Performance scores were scaled to be between 0 and 1 (see methods for details). *H_1_* = *POST < PRE* assessed with Bayesian single-sided paired t-test. Interaction models tested with Bayesian mixed regressions. Estimations made using BayesFactor’s ^4^ default Cauchy prior scale $r=\frac{1}{2} \sqrt{2}$ and prior uniform probability to the models. Approximate classification scheme for the interpretation of Bayes factors from ^5^: *Moderate evidence for *H_1_*,*_,_* **˙** Moderate evidence for *H_0_.*

**References**

1. Salvucci, D. D. & Goldberg, J. H. Identifying Fixations and Saccades in Eye-Tracking Protocols. *Proc. Eye Track. Res. Appl. Symp.* 71–78 (2000). doi:10.1145/355017.355028

2. van der Lans, R., Wedel, M. & Pieters, R. Defining eye-fixation sequences across individuals and tasks: the Binocular-Individual Threshold (BIT) algorithm. *Behav. Res. Methods* **43,** 239–257 (2011).

3. Holmqvist, K. *et al.* *Eye tracking: A comprehensive guide to methods and measures.* (OUP Oxford, 2011).

4. Morey, R. D., Rouder, J. N. & Jamil, T. Computation of Bayes Factors for Common Designs. 54 (2015). at <http://bayesfactorpcl.r-forge.r-project.org/%0ABugReports>

5. Wagenmakers, E. J. *et al.* Bayesian inference for psychology. Part II: Example applications with JASP. *Psychon. Bull. Rev.* 1–19 (2017). doi:10.3758/s13423-017-1323-7

1. Game 3.3, LSAT 02/1992 Prep Test 4; Passage 4.2, LSAT 10/2011 Prep Test 64 [↑](#footnote-ref-1)
2. Game 2.4, LSAT 02/1994 Prep Test 10; Passage 3.2, LSAT 10/1994 Prep Test 12 [↑](#footnote-ref-2)
